# The Quest for Conquering COVID-19: Updates from the Laboratory

**DOI:** 10.1089/pop.2021.0019

**Published:** 2021-02-05

**Authors:** David B. Nash

**Affiliations:** Jefferson College of Population Health, Philadelphia, Pennsylvania, USA.

As I write, a 1-year anniversary that calls for no celebration has arrived. On January 20, 2020, the state of Washington and the Centers for Disease Control and Prevention diagnosed the first US case of the highly contagious virus we've come to know as COVID-19. Sadly, the pandemic death toll (now more than 420,000 Americans) is expected to climb even higher.

For a full year, the urgent needs and complex challenges associated with the pandemic have dominated the news and demanded constant attention from policy makers, the scientific community, and the health care industry. Confronting this new virus has required unprecedented effort on the part of scientists charged with developing and distributing diagnostic tests and vaccines, and frontline clinicians responsible for saving lives.

Although many hurdles have been cleared, other issues have yet to be resolved. For instance, we still don't have tests appropriate for population screening purposes that could be administered at points of care (eg, schools). What we need *today* is a reasonably priced, easily accessed antigen test that can be self-administered with results in minutes rather than days.

We also need what only science can provide – ongoing research that constantly expands our understanding of this novel organism, improves our ability to manage its spread, and helps reduce the propagation of misinformation.

Last year, it came to my attention that Quest Diagnostics, a large American clinical laboratory, had taken a leading role in the global fight against COVID-19. In my estimation, their scientific and research staff rival that of many top universities and their work likely will have a positive impact on combatting this and future outbreaks. The collection of reports and analyses contained in this supplement provide useful point-in-time analyses and insights into effective testing, population screening strategies, and interventions for the near future.

The monumental task of getting Americans back to work will fall squarely on the shoulders of the nation's employers, most of whom are woefully unprepared for this role. Here, Quest is emerging as a national leader in helping to guide employer-based return to work strategies that incorporate the best available evidentiary-based recommendations. Two of the articles in this supplement focus on the evolving role of employers in the longer-term national response to the pandemic.

1. In their article, *Return to Work: Managing Employee Population Health During the COVID-19 Pandemic,* Fragala et al discuss how employers have responded to the health crisis by means of transforming benefits plans to address immediate needs and introducing targeted strategies such as ongoing testing/surveillance and workplace modifications to keep workforces healthy.
2. Plantes et al present an overview of the peer-reviewed literature in their article, *Model for Mitigation of Workplace Transmission of COVID-19 Through Population-based Testing and Surveillance.* They describe an approach that uses a biometric platform informed by real-time PCR test data at the county and subcounty levels. Bioinformatics tools can be used by large employers to mitigate the impact of the pandemic on their workplaces.

The rapid spread of COVID-19 has focused attention on the need for safe, convenient, timely, and scalable methods for collecting respiratory specimens for testing. In their article, *Evolution of Specimen Self-Collection in the COVID-19 Era: Implications for Population Health Management of Infectious Disease*, Cockerill et al discuss the current and future state of self-collection for infectious agents and the impacts of these approaches on population health management, disease diagnosis, and prevention. For instance, self-collection will help prevent the spread of the disease by eliminating the need for physical contact between the patient and health care providers.

The jury is still out on the question of whether and how long an immune response persists following infection with COVID-19. *Insights from Patterns of SARS-CoV-2 Immunoglobulin G Serology Test Results from a National Clinical Laboratory, United States, March-July 2020* points toward a possible answer. Based on their assessment of patterns of immunoglobulin G (IgG) positivity over time in individuals tested for SARS-CoV-2 ribonucleic acid or IgG positivity (more than 2.4 million SARS-CoV-2 IgG serology and 6.6 million acid amplification test results), Kaufman et al draw some conclusions about how some individuals may be infecting household members.

The breadth of the pandemic's ripple effect will be revealed over time. Niles et al have uncovered some disturbing trends stemming from the simultaneous opioid epidemic and COVID-19 pandemic. *The Opioid Epidemic Within the COVID-19 Pandemic: Drug Testing in 2020* describes their analysis of changes in clinical drug testing patterns and results. They found a decrease in overall drug testing with increased positivity for high-risk drugs and dangerous drug combinations – patterns that, if persistent, will lead to an increased need for health care and public health resources dedicated to supporting vulnerable patients and addressing causes.

Conquering COVID-19 will require many more combatants over an extended period of time but, as exemplified in these reports, Quest Diagnostics is helping to lead the charge!

